# Irrigation of the Stomach in the Treatment of Incoercible Hiccough

**Published:** 1894-04

**Authors:** 


IRRIGATION OF THE STOMACH IN THE TREATMENT INCOERCIBLE OF HICCOUGH.—Among the various means of treatment recommended to relieve persistent hiccough—the most recent of which is pilocarpine—no reference is made to irrigation of the stomach. Dr. Brown (Decatur), however, has had recourse to this method with success in the case of a man suffering from incoercible hiccough.

Two other cases of this affection which had resisted all other means of treatment, have recently been reported by Drs. A. Gallant (New York) and P. Coleman (Colorado), in which the intolerable distress was also relieved by washing the stomach.

Dr. Gallant's patient was a robust man who had been suffering for the previous three days from persistent hiccough occurring at intervals of two or three minutes, except during sleep. Temporary relief was obtained first with hydrochlorate of cocaine (in doses of one-tenth of a grain every three hours), then with hydrochlorate of pilocarpine (one-twelfth of a grain three times a day), but the hiccough ultimately reappeared with greater intensity than before. The patient complaining of pain in the epigastrium with a history of dyspepsia, it occurred to Dr. Gallant that the spasms were of gastric origin, and the patient's stomach having been washed the hiccough immediately disappeared.

In Dr. Coleman's case the patient was a man usually in the enjoyment of good health, who had been seized a few days before with incessant hiccough which had resisted the administration of chloral, opiates, bromides, ether and musk. When first seen he was very weak and drowsy from the effects of large doses of the various narcotics above enumerated. The bladder being distended with urine, a catheter was passed, after which the hiccough disappeared for eight hours. It returned at the end of that time, however, as bad as ever. In view of the failure of narcotics to afford relief and of the furred condition of the patient's tongue, Dr. Coleman was led to suspect that the affection in question was due to the presence of gastric troubles, irrigation of the stomach being followed by the complete disappearance of the hiccough.

Irrigation of the stomach being practically a very innocuous procedure, this method of treatment might advantageously be tried not only in cases of hiccough presumably of gastric origin, but even when this manifestation is the result of disturbance of the nervous or other systems. As a matter of fact, there is no reason why this procedure should not produce a favorable effect in cases of nervous hiccough, by diminishing the reflex irritability of the nervous system a result which was achieved in Dr. Coleman's case by the passage of a catheter.—*Ex.*

